# Complement Immune System in Pulmonary Hypertension-Cooperating Roles of Circadian Rhythmicity in Complement-Mediated Vascular Pathology

**DOI:** 10.3390/ijms252312823

**Published:** 2024-11-28

**Authors:** Hunter DeVaughn, Haydn E. Rich, Anthony Shadid, Priyanka K. Vaidya, Marie-Francoise Doursout, Pooja Shivshankar

**Affiliations:** 1 Center for Metabolic and Degenerative Diseases, The Brown Foundation Institute of Molecular Medicine for Prevention of Human Diseases, UTHealth-McGovern Medical School, Houston, TX 77030, USA; devaughn.h166523@stu.sanjac.edu (H.D.); haydn.e.rich@uth.tmc.edu (H.E.R.); anthony.shadid@bcm.edu (A.S.); priyanka.k.vaidya@uth.tmc.edu (P.K.V.); 2 Center for Immunology and Autoimmune Diseases, The Brown Foundation Institute of Molecular Medicine for Prevention of Human Diseases, UTHealth-McGovern Medical School, Houston, TX 77030, USA; 3 Department of Anesthesiology, Critical Care and Pain Medicine, UTHealth-McGovern Medical School, Houston, TX 77030, USA; marie-francoise.doursout@uth.tmc.edu

**Keywords:** complement immune system, pulmonary arterial hypertension, group classification in pulmonary hypertension, clinical studies on pulmonary hypertension with complement intervention, autoimmune diseases, circadian dysregulation

## Abstract

Originally discovered in the 1890s, the complement system has traditionally been viewed as a “compliment” to the body’s innate and adaptive immune response. However, emerging data have shown that the complement system is a much more complex mechanism within the body involved in regulating inflammation, gene transcription, attraction of macrophages, and many more processes. Sustained complement activation contributes to autoimmunity and chronic inflammation. Pulmonary hypertension is a disease with a poor prognosis and an average life expectancy of 2–3 years that leads to vascular remodeling of the pulmonary arteries; the pulmonary arteries are essential to host homeostasis, as they divert deoxygenated blood from the right ventricle of the heart to the lungs for gas exchange. This review focuses on direct links between the complement system’s involvement in pulmonary hypertension, along with autoimmune conditions, and the reliance on the complement system for vascular remodeling processes of the pulmonary artery. Furthermore, circadian rhythmicity is highlighted as the disrupted homeostatic mechanism in the inflammatory consequences in the vascular remodeling within the pulmonary arteries, which could potentially open new therapeutic cues. The current treatment options for pulmonary hypertension are discussed with clinical trials using complement inhibitors and potential therapeutic targets that impact immune cell functions and complement activation, which could alleviate symptoms and block the progression of the disease. Further research on complement’s involvement in interstitial lung diseases and pulmonary hypertension could prove beneficial for our understanding of these various diseases and potential treatment options to prevent vascular remodeling of the pulmonary arteries.

## 1. Introduction

**Figure 1:**
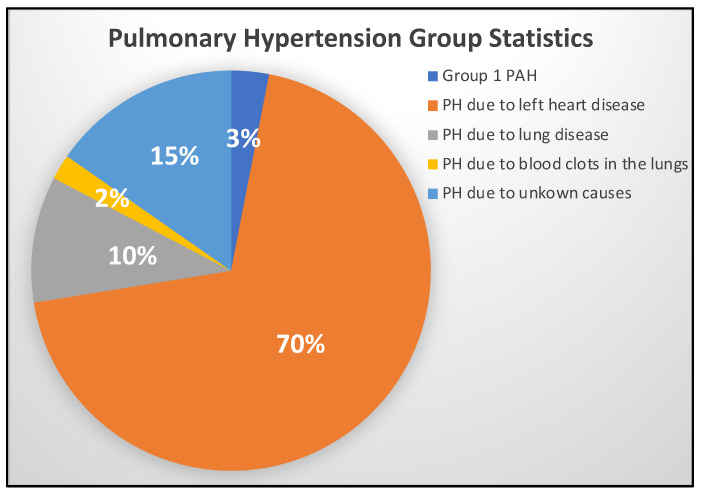
Classification and group statistics of pulmonary hypertension (PH). The Pie chart represents groups of pulmonary hypertension. Note that group 1 pulmonary arterial hypertension (3%) is classified within the broad umbrella of pulmonary hypertension, as it presents normal left arterial pressure and is also referred to as precapillary pulmonary hypertension. PH with left heart conditions is the major incidence in patients (70%), followed by those with unknown causes (15%). Interstitial lung disease-associated PH occurs in about 10% of the PH patients, followed by 2% of PH patients presented with microthrombus formation in the pulmonary arterioles.

Pulmonary circulation begins with the contraction of the right ventricle of the heart to send deoxygenated blood to the lungs for gas exchange, and proper function of blood vessels such as the pulmonary artery is essential for normal pulmonary circulation. Pulmonary hypertension (PH) is a broad term describing chronic high blood pressure of any origin in the pulmonary arteries. In contrast, pulmonary arterial hypertension (PAH) specifically delineates the subset of PH that involves disease and narrowing of the pre-capillary pulmonary blood vessels [1]. This narrowing of the pulmonary blood vessels increases the work required of the heart to pump deoxygenated blood to the lungs efficiently. Chronic mean arterial pressure elevation leads to right ventricular remodeling and dilatation, interfering with proper gas exchange in the lungs [2,3]. This dilated right ventricle reduces left ventricular filling by occupying more of the limited space within the pericardium, with a subsequent decrease in cardiac output. In concert with increased right-sided filling pressures and reduced right ventricle output, the lowered cardiac output contributes to the common PH symptoms of fatigue, exertional dyspnea, and systemic venous congestion. While the broad classification of PH includes PAH, this subcategory of PH has additional diagnostic requirements. PH is characterized simply by a mean arterial pressure greater than 25 mmHg at rest. In comparison, a diagnosis of PAH requires a pulmonary capillary wedge pressure of 15 mmHg or less in the context of a pulmonary vascular resistance of more than 3 wood units (mmHg/L/min) [4,5]. Globally, the prevalence of PH is estimated to be around 1%, with roughly 49–55 cases of PAH per one million adults [4,6]. PAH is considered rare, with a relatively poor prognosis and an average life expectancy after diagnosis of 2–3 years in subtypes such as idiopathic pulmonary arterial hypertension (IPAH) [7,8]. Common risk factors associated with PH include poor physical health, connective tissue diseases, and other heart- and lung-related vascular diseases; however, the direct causes of PH are still not fully understood. It is common for PH to go undiagnosed in patients for months or even years, because the signs and symptoms closely resemble those of other heart conditions and often develop slowly over time [9,10,11]. Five major groups with distinct clinical presentations and pathologies comprise the broader classification of PH. One study that examined just over 4500 patients with PH found that the most common clinical presentation of PH was that which arose due to left heart disease (group 2), followed by that in patients with lung diseases (group 3). The third leading cause of PH was group 5 or idiopathic PH. Together, PAH (group 1) and PH due to thrombus or embolus-associated lung disease (group 4) accounted for less than 5% of the PH cases investigated [1] (Figure 1). While there is currently no cure, therapeutic treatments for PH have been available since 1995, and continual advancements help treat patients and identify biomarkers with which to detect the disease early in its development [8,10,11,12,13]. There are currently five different drug classes available to treat PAH: calcium channel blockers (CCBs), endothelin receptor antagonists (ERAs), phosphodiesterase type 5 inhibitors (PDE-5i), soluble guanylate cyclase stimulators (sGCSs), and prostacyclin analogs (PCAs) [14,15]. In this review, we discuss the role of complement immunity in PH pathogenesis and how future research on complement could enhance our understanding of PH.

## 2. The Complement System

**Figure 2:**
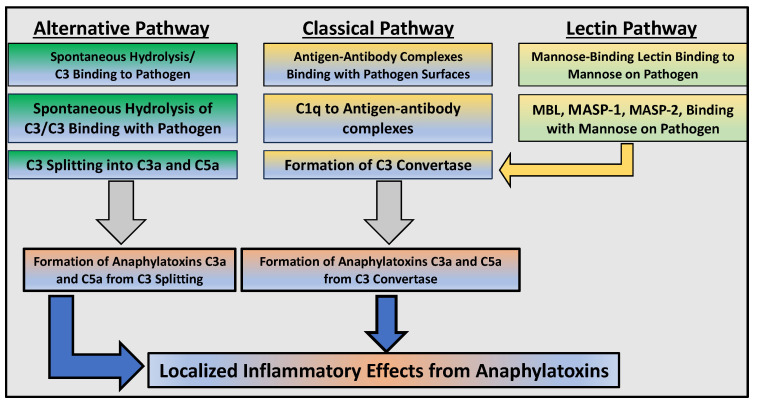
Schematic illustration of complement immune cascades. The three common cascades of complement activation are the alternative, the classical, and the lectin pathways. The alternative pathway is considered the surveillance pathway that is activated spontaneously during cell injury and pathogenic assaults with the complement 3 (C3) hydrolysis, leading to potent inflammatory peptides formations called C3a and C5a anaphylatoxins. These anaphylatoxins are known to sustain and exacerbate inflammatory responses via their cognate receptors C3aR- and C5aR-activated proinflammatory signaling. The classical pathway, as the name suggests, is activated via the binding of complement 1 subunits complex (C1-qrs) to the circulating antigen–antibody complexes formed during tissue damage and pathogenic assaults. This leads to activation of the complement cascade via C2, C3, and C4 splitting and the formation of C3a and C5a anaphylatoxins. The lectin pathway is triggered by the cell surface-bound mannose residues, binding to the soluble mannose-binding lectins in the host, resulting in anaphylatoxin production. After converging at the level of C3 convertase and the formation of C3a and C5a anaphylatoxins, the three cascades proceed with the formation of the membrane attack complex (MAC), also known as the terminal complement complex (TCC), comprising C5b–C6–C7–C8 and 12–18 copies of C9 (C5b–C9 complex). While MAC serves as the significant effector of the innate immune system that disrupts cell membranes, leading to cell death, C3aR and C5aR signaling is involved in integrating an adaptive immune response by producing chemotactic mediators.

The complement immune system has been linked to the pathogenesis of PH. It consists of over 30 blood plasma proteins formed in the liver that play various roles in the body’s immune function. The complement system cascade is activated by three pathways: classical, alternative, and lectin. Different reactions activate each complement pathway, but all involve cleavage of the C3 complement protein into C3a and C3b by C3 convertase. The classical pathway activates when C1q reacts to immune complexes by binding the Fc region of immunoglobulins IgG and IgM [16]. This triggers a cascade in which the subsequent cleavage of proteins C2 and C4 forms C3 convertase. Activated C3 convertase splits C3 into anaphylatoxin C3a and opsonin C3b, which further amplifies immune response and contributes to the formation of C5 convertase. Other relevant proteins include the anaphylatoxin C5a and proteins C5b–C9, which form the membrane attack complex (MAC). The lectin pathway is activated by the binding of mannose-binding lectin (MBL) or ficolin to the surface of pathogens; this induces the activation of MBL-associated protein 2 (MASP2), which triggers a series of cleavages that also ultimately produce C3 convertase. In the alternative pathway, basal levels of C3 hydrolysis occur constantly. C3b binds to nearby bacteria and recruits both Factor B and D to form a C3 convertase [17,18,19] (Figure 2). Complement mediates the destruction of pathogens, attraction of leukocytes, and inflammatory processes [20].

One of the main functions of the complement system is the regulation of localized tissue inflammation. Inflammation helps the body defend against pathogens and repair injured tissue, but it damages tissue when chronically or inappropriately stimulated [21]. While most complement proteins are produced in the liver and reach effectors via circulation through the bloodstream, the airway epithelium can also produce complement proteins locally [22]. Numerous human subject-based and experimental studies show causative links and correlations between local complement protein production and various chronic inflammatory and autoimmune conditions affecting the lungs, such as systemic lupus erythematosus, dermatomyositis/polymyositis, rheumatoid arthritis, and systemic sclerosis with PAH and interstitial lung disease (ILD) manifestations. As the focus of this review, PH and ILDs, chronic obstructive pulmonary disease (COPD), and idiopathic pulmonary fibrosis (IPF) will be further discussed [23,24].

## 3. Complement in Biochemical Processes of Vascular Remodeling in PAH

Vascular remodeling is a dynamic process where physical and chemical changes alter the structure of blood vessels. Blood vessel walls consist of three separate layers: the tunica intima, tunica media, and tunica adventitia [25]. Different cell types, including endothelial cells, smooth muscle cells, and fibroblasts, comprise these layers. The physical changes involved in vascular remodeling due to hypertension include endothelial cell disruption in the tunica intima, smooth muscle cell hypertrophy in the tunica media, and fibroblast stimulation in the tunica adventitia. Vascular remodeling of the pulmonary artery narrows the lumen diameter, resulting in chronically increased blood pressure. As the luminal diameter of a blood vessel shrinks, blood pressure increases, stimulating more vascular remodeling [26,27,28,29,30]. Furthermore, a positive feedback loop can reinforce and exacerbate existing vascular remodeling [31]. Chronic localized inflammation is linked to arterial stiffening and vascular remodeling processes [32].

The tunica intima comprises endothelial cells that regulate vascular permeability and coagulation responses. These endothelial cells contain receptors for multiple ligands, including complement proteins C3a and C5a. Various cells in the body, including mast cells, macrophages, epithelial cells, and endothelial cells, have receptors for anaphylatoxins. The binding of anaphylatoxins on vascular endothelial cells elicits many pro-inflammatory responses, including the activation and attraction of lymphocytes, release of cytokines, and attraction of other immune cells [33]. Recent studies have demonstrated that endothelial cells stimulated with C3a and C5a release various inflammatory cytokines such as interleukin 1-alpha (IL-1α), interleukin 6 (IL-6), and the chemokine interleukin 8 (IL-8). IL-1α acts on various cells involved in inflammatory responses, such as macrophages, fibroblasts, and endothelial cells [33,34]. IL-1α acts via downstream intracellular signaling and has gene transcription effects on many genes, including its own [34,35]. Increased production of IL-6 is heavily associated with chronic inflammation and various autoimmune disorders; it acts in localized inflammatory responses by regulating acute-phase pro-inflammatory proteins [36,37]. Dysregulation of this process facilitates chronic inflammation [38,39]. IL-8 is another chemokine involved directly in localized acute inflammatory reactions via its role in the activation and attraction of neutrophils [40,41,42].

Thickening of the tunica media layer containing vascular smooth muscle cells (VSMC) is a hallmark pathophysiological mechanism in the vascular remodeling seen in PH. Chronic biomechanical stress, such as increased contractions, stimulates vascular smooth muscle cell hypertrophy [27,43,44,45]. Anaphylatoxins mediate smooth muscle cell contraction and contribute to this process [46]. Vascular smooth muscle cells also contain receptors for interleukin-6 (IL-6R). While research on this mechanism is limited, IL-6 has been shown to act on VSMCs to stimulate hypertrophy [47,48].

Anaphylatoxins can bind to target cells via the anaphylatoxin receptors C3aR and C5aR to modulate inflammation. Target cells containing anaphylatoxin receptors C3aR and C5aR include endothelial cells, mast cells, macrophages, and other leukocytes [36,37]. Macrophages, mast cells, and fibroblasts are all prevalent within the tunica adventitia, the outermost layer of blood vessels. Upon activation from anaphylatoxins, mast cells within the tunica adventitia release chemicals such as histamine, tumor necrosis factor (TNF), and other pro-inflammatory cytokines. The release of these chemicals by mast cells promotes localized inflammatory effects and attracts neutrophils and macrophages [33,49,50,51,52]. The binding of anaphylatoxins to macrophages triggers the upregulation and release of the cytokine oncostatin M. Its effects include the promotion of inflammation and remodeling of the extracellular matrix. While data on oncostatin M and anaphylatoxins are limited, this is potentially one of the direct mechanisms for complement’s involvement in localized inflammation and tissue remodeling [53].

**Figure 3:**
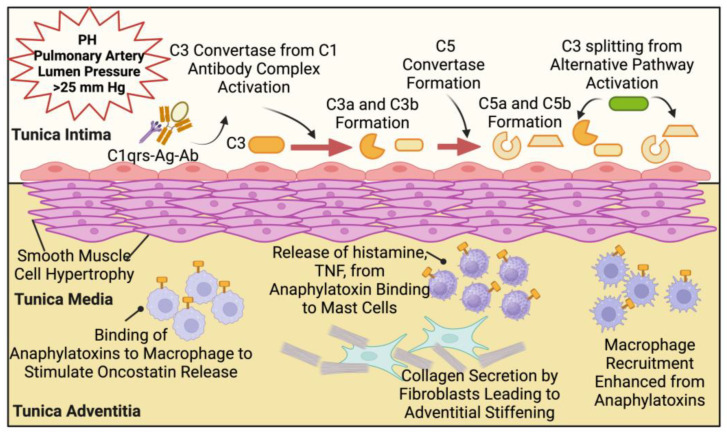
Complement-associated biochemical interactions in the pathogenesis and tissue remodeling in PH. Both classical and alternative complement pathways are associated with the modulation of inflammation and tissue remodeling. Antigen–antibody complexes of IgM and IgG conjugating with C1qrs trigger classical pathways and endothelial cell inflammation by C3aR/C5aR-mediated signaling, thereby promoting smooth muscle cell hypertrophy and immune cell infiltration. While the binding of C3a/C5a to their receptor mast cells exacerbate inflammation by mediators such as histamine and TNF, C3aR/C5aR signaling in macrophages lead to the production of potential remodeling mediators such as oncostatin M, resulting in myofibroblast activation and sustained recruitment of inflammatory macrophages.

IL-6 also directly affects fibroblasts and stimulates their differentiation into myofibroblasts by mediating intracellular signaling pathways [54]. Myofibroblasts secrete increased amounts of collagen type 1 and enzymes, which enhance the formation of the extracellular matrix [55]. Emerging research has linked IL-6 with vascular remodeling of blood vessels through its involvement in localized inflammation. A study recently completed in 2023 directly linked IL-6 with the development of atherosclerosis and vascular remodeling [56]. Stimulation of effector cells by complement protein anaphylatoxins enhances inflammatory processes that contribute to vascular remodeling of the pulmonary arteries. Given that mast cells, fibroblasts, and macrophages are prevalent within the tunica adventitia, this could imply that vascular remodeling occurs on multiple fronts in the development of PH. Nevertheless, isoliquiritigenin, a flavonoid of licorice root (*Glycyrrhizae radix*), attenuated both hypoxia-induced PH and monocrotaline (MCT)-induced PH in mice by significantly inhibiting *Il6* production and pulmonary arterial smooth muscle cell proliferation [57,58]. More importantly, C3-deficient transgenic mice exhibited a decreased severity of hypoxia-induced PAH, with significant downregulation of the *Il6* and intercellular adhesion molecule-1 (*Icam-1*) levels. They also demonstrated abrogation of prothrombotic mediators; the tissue factor, also known as coagulation factor III; and fibrin deposition [59]. Collectively, these studies suggest a direct role of complement in PAH-associated vascular tissue remodeling. Given that C3 is the convergent point of all three complement cascades, leading to C3aR and C5aR anaphylatoxin receptors signaling, the potential targeting of C3 is a current goal in the development of complement-based therapeutics for treating chronic inflammatory and autoimmune diseases [60]. Figure 3 outlines the potential biochemical processes involved in the PH pathogenesis and tissue remodeling modulated by complement and anaphylatoxins-mediated signaling.

## 4. Complement Involvement in Pulmonary Arterial Hypertension

The complement system plays a significant role in the development and progression of PAH by perpetuating inflammation and promoting vascular remodeling. Research has demonstrated that complement activation, particularly involving the C3 component, contributes to the inflammatory processes that underlie PAH. In studies on chronic hypoxia-induced PAH, C3 deficiency in animal models was associated with reduced pulmonary artery pressure and decreased vascular remodeling, suggesting that C3-driven complement activation exacerbates PAH by promoting immune cell recruitment and inflammation in the pulmonary vasculature [59]. These findings indicate that C3 and the broader complement system, when dysregulated, prove instrumental in advancing vascular changes characteristic of PAH. This also positions complement components as potential therapeutic targets to mitigate disease progression.

Within the complement system, the classical pathway specifically contributes to PAH by driving structural changes in the pulmonary arteries. Activation of C1q, the initiating component of the classical pathway, has been shown to activate β-catenin signaling in vascular smooth muscle cells. This signaling pathway promotes smooth muscle cell proliferation and arterial thickening, both critical features of hypertensive vascular remodeling. Such remodeling contributes to increased arterial stiffness and heightened vascular pressure, which are central to PAH pathology. By linking C1q-induced activation with β-catenin-mediated cell proliferation, this pathway represents a mechanism by which the classical complement pathway exacerbates vascular remodeling in PAH [61]. These insights suggest that targeting the classical pathway, particularly the C1q-β–catenin axis, may provide new therapeutic approaches to address the maladaptive vascular changes that drive PAH.

The alternative pathway of the complement system plays a critical role in amplifying inflammatory responses and promoting vascular remodeling in PH. Studies have shown that alternative pathway activation, particularly in autoimmune contexts such as lupus nephritis, correlates with elevated pulmonary artery pressures and enhanced inflammatory markers [62]. In PH, alternative pathway activation is often driven by immunoglobulin deposition in the pulmonary arteries; this initiates a complement cascade that recruits immune cells, stimulates cytokine production, and fosters an inflammatory environment conducive to vascular remodeling [63]. Once activated, the alternative pathway acts as an amplification loop, intensifying the production of key complement components like C3b and promoting terminal complement activation [64]. This feedback mechanism not only exacerbates immune cell recruitment and inflammatory signaling but also drives smooth muscle cell proliferation and fibroblast activation, leading to arterial thickening and increased rigidity. By sustaining and magnifying the inflammatory responses initiated by the classical pathway, the alternative pathway emerges as a crucial driver of complement-mediated pathology in PH, highlighting its potential as a therapeutic target for reducing vascular inflammation and remodeling in the disease [65].

Furthermore, pulmonary arteries reflexively constrict within minutes of exposure to hypoxic environments to divert blood to regions of the lung with better oxygenation [66]. The study examined human lung specimens from PAH patients, as well as from murine models with hypoxia-induced PAH. Local complement protein production and activation were confirmed in the hypoxia-induced PAH mice and human patient lung samples via immunostaining for deposited complement protein C3 [65]. Within minutes of murine model exposure to hypoxic environments, complement protein C3 deposition occurred. Researchers also observed increased numbers of cells with anaphylatoxin receptors C3aR and C5aR throughout the tunica adventitia. The findings are significant, as they show a link between the complement system cascade and hypoxia-induced PH.

This study also analyzed whether the complement system was directly involved in modulating the chemotaxis of immune cells. Researchers used mice with a genetic complement deficiency, and after immunostaining of lung sections obtained from these mice for CD68 macrophage markers, a significantly lowered macrophage recruitment was confirmed. This indicates that the complement system directly enhances macrophage recruitment, a notable contributor to localized tissue inflammation. It was also discovered that these mice had much lower levels of pro-inflammatory cytokines when compared to those under normal oxygen conditions.

IgG and IgM serum antibodies in the bloodstream can activate the classical pathway of the complement system under chronic inflammation [67,68]. A third goal of this study was to determine if there was a link between hypoxic environments, classical pathway complement system activation, and vascular remodeling processes. After murine models were exposed to hypobaric environments to induce hypoxic PH, researchers noted an upregulation of immunoglobulins. IgM deposition occurred primarily in the tunica intima and tunica media layers, while IgG deposition occurred primarily in the tunica adventitia and was accompanied by complement protein C3. The control group of mice under normal oxygen conditions did not show any deposition of antibodies or C3.

Murine models genetically modified to exhibit deficiencies in IgG and IgM were protected from hypoxia-induced vascular remodeling and did not display signs of hypoxia-induced inflammatory responses associated with activation of the complement cascade. IgG-deficient mice were injected with sufficient IgG to reach normal levels to further validate that the immunoglobulin presence directly correlated with hypoxia-induced responses such as macrophage recruitment, cytokine production, and other pro-inflammatory responses. After the IgG levels increased, the mice displayed signs of complement activation, enhanced macrophage recruitment, and pro-inflammatory responses associated with vascular remodeling [65].

Although most of the data come from lung tissue in mouse models, the anatomy and physiology of lungs in mice are similar to that of humans. Some differences exist between mouse and human lung tissues, such as the proportion of neutrophils and receptor types, as well as notable differences in the respiratory rate and physical size of the lungs [69]. Murine models have proven useful for immunological and respiratory research and have been used to make important discoveries of major histocompatibility complex (MHC) activities, gene transcription processes, and antibody synthesis mechanisms.

## 5. Complement Involvement in Group 3 Pulmonary Hypertension

Group 3 PH, the second-most common subtype of PH, is caused by chronic lung diseases such as (COPD), pulmonary fibrosis, and ILDs [70]. Over time, these chronic lung diseases can cause hypoxia and increase blood pressure within the pulmonary arteries. There is increasing evidence linking the complement system with the pathogenesis of lung diseases [24,71].

COPD is characterized by chronic airflow obstruction in the lungs that inhibits breathing and creates a hypoxic environment [72]. This disease is commonly associated with group 3 PH development. As previously stated, pulmonary arteries constrict in response to ventilation/perfusion mismatch, so blood can divert to locations that maximize oxygenation. Chronic inhalation of cigarette smoke is a major risk factor for the development of COPD, and tobacco glycoproteins have been shown to activate complement pathways by binding with C1q and increasing the production of C5a [73]. Tobacco glycoproteins activate the complement classical pathway in vivo by their direct binding site for complement C1q [73]. Chronic inhalation of tobacco glycoproteins leads to the localized activation of complement pathways within the lungs and airway could explain some of the underlying reasons for the elevated risk of COPD development in chronic smokers. As previously discussed, anaphylatoxin C5a is involved in various inflammatory reactions due to its binding of C5aR1 receptors on target cells such as fibroblasts, mast cells, and macrophages [74]. Tumor necrosis factor is an inflammatory cytokine that macrophages produce and release upon the binding of anaphylatoxins [50]. It is a pro-inflammatory cytokine linked to COPD, lung cancer, and other lung diseases when overexpressed [75]. Future research on tumor necrosis factor release due to anaphylatoxin action could provide useful information to understand PH due to lung disease.

Pulmonary fibrosis is a disease characterized by thickening and scarring of the parenchyma and alveoli in the lungs and contributes to some cases of type 3 PH. Over time, the fibrosis leads to chronic inhibition of the airflow [76]. Recent data have shown a high prevalence of complement protein C1q of the classical pathway in patients with pulmonary fibrosis [77]. As previously discussed, IL-6 promotes the fibroblast to myofibroblast phenotype change through various intracellular signaling pathways. Increased numbers of myofibroblasts are often observed in patients with pulmonary fibrosis [78]. Given that myofibroblasts secrete collagen and accentuate extracellular matrix (ECM) deposition, they likely play a role in pulmonary fibrosis [79].

## 6. Complement System in Autoimmune and Inflammatory Diseases

### 6.1. Systemic Lupus Erythematosus

The complement system plays a central role in the pathophysiology of many autoimmune and inflammatory diseases by promoting inflammation, immune cell recruitment, and vascular remodeling. In systemic lupus erythematosus (SLE), for instance, complement deficiencies in components like C1q, C4, and C2 impair the clearance of immune complexes, leading to immune dysregulation and chronic inflammation [80]. This dysfunction not only contributes to disease progression but also exacerbates tissue damage, particularly in organs like the kidneys. Additionally, anti-C1q antibodies present in many SLE patients further activate the classical pathway, driving inflammation and tissue damage, especially in lupus nephritis [81]. The updated classification criteria for SLE underscore complement components as biomarkers for disease activity, highlighting low C3 and C4 levels as indicators of active disease, particularly in patients with severe organ involvement [82]. Sugimoto et al. (2017) have demonstrated the link between autoimmune disease and pulmonary hypertension using *MRL/Lpr* mice that develop hypergammaglobulinemia, which results in spontaneous vasculitis and nephritis, mimicking SLE disease presentation. These mice displayed IgG and C3 deposition in their kidneys and significantly elevated the serum interferon-gamma (Ifn-γ) and Il-6 levels, along with right ventricular (RV) systolic pressure, right ventricular hypertrophy measured as the Fulton index, ratio of right ventricular weight to the sum of left ventricular (LV) and septal (S) weights (Fulton index = RV/LV + S), and ratio RV weight to whole body weight in comparison to those of wild-type C57BL/6 mice [83,84].

### 6.2. Dermatomyositis/Polymyositis

Characteristics of interstitial lung diseases and pulmonary arterial hypertension are also evident in dermatomyositis (DM) patients. Given that DM involves complement-mediated microangiopathy the inactive C4 fragmented peptide, C4d is predominantly seen in the muscle biopsy samples of these patients, as well as in nonspecific myositis patients, as a marker of complement activation and membrane attack complex (MAC) formation [85]. It is noteworthy that the C4 and IL-6 levels were significantly elevated in the plasma of patients with DM associated with interstitial lung disease, chronic inflammation, and PAH presentation from the non-complicating DM. These patients also showed significantly increased lymphocytic burden with T helper cells, natural killer cells, and B lymphocytes [86]. At the molecular level, classic complement factors C1QB and C1QC were significantly upregulated in the plasma exosomes of DM and polymyositis (PM) patients compared to those isolated from healthy control plasma, implicating the complement in the immunopathogenesis of DM/PM as a mediator of microvascular damage and muscle weakness [87].

### 6.3. Rheumatoid Arthritis

Complement dysregulation is similarly involved in other autoimmune diseases, such as rheumatoid arthritis (RA) and systemic sclerosis (SSc). In RA, the complement system interacts with inflammatory cytokines and vascular growth factors, creating a pro-inflammatory environment that promotes endothelial dysfunction and synovial remodeling [88,89]. However, RA patients can have severe functional limitations that prevent the early diagnosis of PH with the pulmonary manifestation of RA and therefore predispose it to right ventricular failure. Patients with rheumatoid arthritis-associated interstitial lung disease (RA-ILD) may lack detectable serum anti-citrullinated protein antibodies (ACPA). Similarly, thromboembolic disease and vasculitis with or without the presence of anti-neutrophil cytoplasmic antibodies (ANCA) may also corroborate with the incidence of PH in these patients, warranting the early detection of RA-ILD patients and successive combinatorial treatment for PH to prevent heart failure [90,91,92]. Moreover, in ANCA-associated vasculitis (AAV), the alternative pathway amplifies inflammation and contributes to vascular injury in RA patients. Complement components like C3 and C5a drive immune cell recruitment and endothelial damage, linking complement activation to chronic inflammation and vessel remodeling [93]. In these diseases, complement activation triggers a cycle of immune cell recruitment, endothelial damage, and fibrosis, leading to vascular remodeling, along with thrombosis, and thereby highlighting the potential for complement-targeted therapies [94,95]. A clinical study named SAPHIRE (Stress and Pulmonary Hypertension in Rheumatoid Evaluation) reported a significant association between PH and RA. It evaluated patients with exercise echocardiographic (EchoCG) analysis, in which participants demonstrated significantly high pulmonary arterial pressure (PAP), implicating RA and similar autoimmune conditions as significant risk factors for PAH [96].

### 6.4. Systemic Sclerosis

Systemic sclerosis is a chronic disease characterized by systemic hardening of the skin, internal organs, and blood vessels. The cause of systemic sclerosis is currently unknown. However, multiple theories propose infections, environmental toxins, and immune dysregulation as possible causes. Endothelial cell disruption of the tunica intima is a feature of systemic sclerosis that leads to disturbed blood flow and further stimulation of vascular remodeling due to chronic elevated blood pressure [97]. A study completed in 2022 examined blood samples from systemic sclerosis patients through complement hemolysis, a screening assay that provides a measurement of activation of the classical pathway of the complement system [98]. Evaluation of these samples revealed significantly higher levels of complement proteins relative to healthy controls. In vitro, human umbilical vein endothelial cells (HUVECs) were stimulated with scleroderma-specific antibodies to determine the pro-inflammatory effects on the endothelium. HUVECs stimulated with these antibodies displayed a significantly elevated production of IL-6, which is heavily associated with chronic inflammation and various autoimmune disorders due to its modulatory effect on the production of acute phase pro-inflammatory proteins [47,99]. As previously mentioned, IL-6 directly stimulates fibroblasts’ differentiation into myofibroblasts by mediating intracellular signaling pathways. IL-6 also acts directly on vascular smooth muscle cells to stimulate smooth muscle cell hypertrophy, a mechanism seen in vascular remodeling [48].

Interestingly, SSc patients also exhibit mannose-binding lectin (*MBL*) and *ficolin-2* (*FCN2*) polymorphisms with significant elevation in the serum levels of ficolin-2 and MBL, the pattern recognition receptors of the lectin pathway. The study specifically linked this elevated expression to endothelial damage and fibrosis, both of which are central to vascular complications in this disease [100]. Additional research on complement system activation of endothelial cells to produce pro-inflammatory cytokines and chemokines such as IL-6 could provide more insight into the downstream pro-inflammatory effects of complement activation seen in various disease processes, including PH and SSc.

The complement system’s impact on vascular inflammation and remodeling in autoimmune diseases has significant implications for PAH. As in autoimmune diseases, PAH is characterized by chronic inflammation, immune cell infiltration, and structural changes within the vasculature. Studies in atherosclerosis, a condition marked by vascular inflammation and remodeling, show that complement activation drives plaque formation and endothelial dysfunction [101]. This complement-mediated vascular remodeling may be similarly relevant in PAH, where immune-driven endothelial damage and vascular thickening contribute to increased pulmonary pressure. Additionally, research in vasculitis has demonstrated that mechanisms like necroptosis can lead to neutrophil extracellular trap (NET) formation, which subsequently activates the complement and exacerbates vascular damage [102]. This pathway of immune cell death and complement activation could also be a factor in PAH, as immune cell recruitment and complement activation contribute to the disease’s inflammatory milieu.

**Figure 4:**
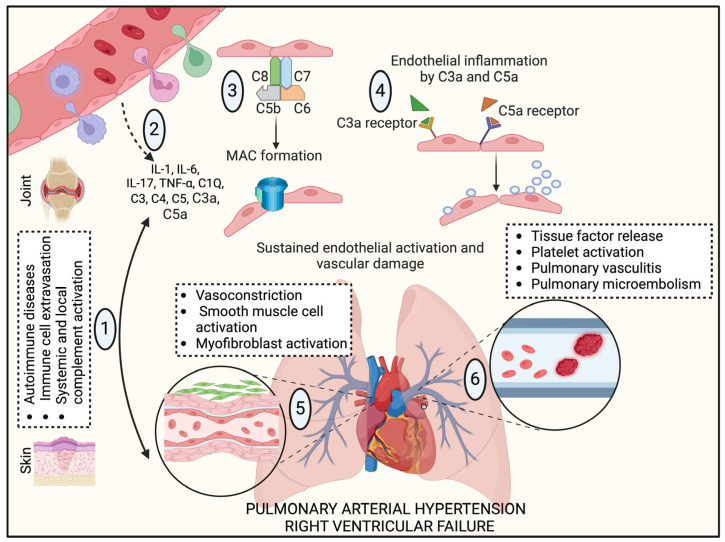
Autoimmune diseases and complement activation in the pathogenesis of PAH. (1) Autoimmune conditions include, but are not limited to, RA (joints), SLE, SSc, and dermatomyositis (skin) that promote inflammatory cell infiltration, enhance systemic and local inflammatory responses, and complement activation. (2) Release of proinflammatory cytokines, including IL1, IL6, and TNFα, directly activates endothelial cells and impairs vascular relaxation, resulting in significant vasoconstriction and smooth muscle cell activation due to reduced endothelial nitric oxide production. (3) The complement components are produced by the liver released in systemic circulation and contributed by infiltrated immune cells resulting in the formation of MAC and, therefore, endothelial injury. (4) C3a/C3aR and C5a/C5aR signaling exacerbate chemotaxis, immune cell extravasation, and platelet activation by the endothelial pro-coagulating tissue factor. (5) Finally, myofibroblast-like vascular smooth muscle cells can contribute to thick vascular media and the production of α-smooth muscle actin and collagen type I in the pulmonary vasculature leading to PAH, and (6) pulmonary vasculitis and micro-emboli formation play significant risk factors in the severity of PAH.

Altogether, the role of the complement system in promoting inflammation, endothelial dysfunction, and fibrosis across various autoimmune diseases offers valuable insights into its potential contributions to PAH. The complement system may be a critical factor in PAH pathology by driving vascular remodeling through persistent inflammation and immune activation (Figure 4). Targeting specific complement pathways could thus represent a promising therapeutic strategy for mitigating vascular damage and managing disease progression in both autoimmune conditions and PAH [103].

## 7. Circadian and Complement in Association with Pulmonary Hypertension

The circadian rhythm is a complex biological process in eukaryotic organisms such as plants, animals, and humans that regulates physical, emotional, and behavioral patterns [104]. Exposure to light and darkness largely influences the circadian rhythm, but several other biological mechanisms have additional impacts [105]. In vertebrate animals such as humans, neurons in the suprachiasmatic nucleus (SCN) are the master regulators behind the organism’s internal circadian rhythm [106]. Transcription factors such as circadian locomotor output cycles protein kaput (CLOCK) and basic helix-loop-helix ARNT-like protein 1 (BMAL1) in the SCN, liver, heart, adipose tissue, and immune cells encode genes such as *PER1*, *PER2*, *CRY1*, and *CRY2*, which regulate the circadian rhythm via a transcriptional feedback loop [107,108,109,110,111]. Dysregulation of the circadian rhythm is linked to the pathogenesis of various diseases, including sleep disorders, neurologic disorders, cardiovascular diseases, and lung diseases [112,113,114].

The circadian rhythm influences thrombogenesis, leukocyte adhesion, and localized inflammation in the vascular endothelium [115]. While research on vascular endothelium and dysregulated leukocyte adhesion remains limited, evidence points to circadian rhythm effects on localized vascular remodeling and vascular smooth muscle cells of the tunica media [116,117]. Peroxisome proliferator-activated receptors (PPARs) are nuclear receptors that function as gene transcription factors. They play a role in regulating BMAL1 and influence vascular smooth muscle cells [118]. Previous studies have identified increased blood pressure in smooth muscle PPAR knockout mice [119]. Fibroblasts, cells prevalent in the tunica adventitia that play a role in localized inflammation and producing collagen, also display susceptibility to circadian clock regulation [120]. Recent studies in 2023 showed the direct effects of circadian gene *BMAL1* expression on lung fibroblasts [121]. *BMAL1* knockdown lung fibroblasts exhibited increased C-X-C motif chemokine ligand 5 (CXCL5) expression when stimulated with interleukin-1 beta (IL-1β). Interleukin-1 beta is a pro-inflammatory cytokine that assists in host immune response and has downstream effects on many cells, including fibroblasts. It stimulates fibroblast to myofibroblast transformation and increases ECM deposition in lung fibroblasts in concert with other cytokines. Interleukin-1 beta has also been shown to impact VSMC directly. In vitro IL-1β levels have also been correlated with PH, as elevated levels of IL-1β are consistently found in PH patients [122]. CXCL5 is a neutrophil chemoattractant that impacts localized inflammatory processes and stimulates various intracellular signaling pathways involving C-X-C chemokine receptor 2 (CXCR2) and signal transducer and activator of transcription 3 (STAT3) [123]. While neutrophils contribute to robust host immune responses, overstimulation of neutrophil recruitment can damage tissue [124]. Research on this mechanism is currently limited, but there are studies that link neutrophil recruitment to vascular remodeling. Mice lung tissue with suppressed neutrophil recruitment exhibited far less vascular remodeling than lung tissue with enhanced neutrophil recruitment [125].

Studies show that mice deficient in circadian clock genes *CRY1* and *CRY2* are more prone to autoimmune disorders and exhibit elevated IgG and IgM serum concentrations [126]. PAH is a common complication associated with autoimmune conditions in humans, and one function of IgG and IgM antibodies is the activation of the classical pathway of the complement system [67]. In preclinical studies, mice deficient in the *CRY1* and *CRY2* genes exhibit decreased levels of complement regulatory proteins such as protectin (CD59) and decay accelerating factor (DAF) [127]. CD59 protects host cells from destruction by the MAC formed by complement proteins C5–C9 [128]. Dysregulation of the complement system and complement regulatory proteins correlates well with inflammation and disease pathogenesis [129,130]. The complement system also plays a role in thrombosis, as platelets display receptors for complement component C1q and complement regulatory proteins such as CD55 and CD59 [131,132,133]. While further research is necessary to draw conclusions, it is possible that dysregulated complement could contribute to group 4 PH via dysregulation of platelets and subsequent thrombosis formation. The circadian rhythm also affects platelet function, and thrombosis and perturbances could initiate or exacerbate platelet dysfunction [134]. Notably, evidence shows that dysregulation of the *CLOCK* and *BMAL1* genes directly impacts megakaryocyte function and the von Willebrand factor (vWF), leading to a prothrombotic phenotype [135].

**Figure 5:**
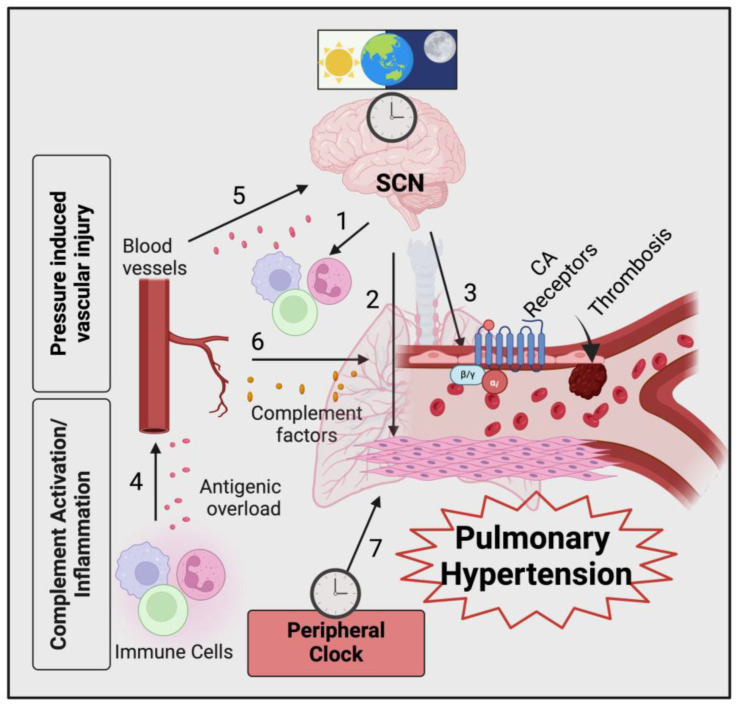
Potential overlapping events of aberrant circadian and complement associated with pulmonary hypertension. Aberrant circadian rhythmicity with impaired CLOCK/BMAL functions in the suprachiasmatic network (SCN) may induce increased proinflammatory cytokines production, thereby enhancing systemic inflammation (1) and pulmonary vascular remodeling contributed by local endothelial cell and smooth muscle cell activation (2,3). Complement immune effectors modulate chronic inflammation by complexing with the antigenic overload and subsequent release of proinflammatory mediators, including C3a and C5a (4–6), thereby cooperating with both central circadian and peripheral clock (7) deflection, leading to the progression of pulmonary hypertension.

The circadian rhythm also plays a role in developing cardiovascular and lung diseases such as COPD and asthma [136]. Studies in mice have shown that increased secondhand smoke exposure causes dysregulated *BMAL1* and *CLOCK* gene expression [137]. Intracellular adhesion molecule 1 and vascular adhesion molecule 1 (VCAM-1) both function to regulate leukocyte trafficking and adhesion to the airway epithelium and are downregulated by perturbances in the circadian rhythm [138]. Dysregulation of ICAM-1 and VCAM-1 is linked to various inflammatory and cardiovascular disorders [139]. Dysregulation of the CLOCK and BMAL1 genes occurs in lung and heart chronic diseases that can precipitate PH groups 2 and 4 [140]. While further research on the circadian rhythm, the complement system, and biochemical processes related to disease pathogenesis is needed, it is likely that these mechanisms lead to the development of inflammation and disease pathogenesis and will prove useful in improving treatments for and prevention of PH (Figure 5).

## 8. Current Treatments for Pulmonary Hypertension

Eculizumab is a complement system inhibitor used in the treatment of various diseases, such as myasthenia gravis and paroxysmal nocturnal hemoglobinuria. Eculizumab is a recombinant human monoclonal antibody that binds directly to complement protein C5 and inhibits cleavage to C5a and C5b [141]. While data on eculizumab are limited, it has been shown to improve hypertension and recovery rates in patients with hemolytic uremic syndrome (HUS), a disorder commonly treated with eculizumab [142]. Pulmonary hypertension is a complication associated with nearly all forms of anemia, including HUS. Various biochemical processes, such as endothelial cell disruption, nitric oxide depletion, and oxidative stress, all contribute to PH, as seen in HUS [143,144]. In the setting of HUS, eculizumab enacts a terminal complement blockade and attenuates inflammation [131]. Pulmonary vascular resistance showed improvement among patients treated with eculizumab, documented by lowered levels of N-terminal pro-brain natriuretic peptide (NT-proBNP), a marker of pulmonary vascular resistance and right ventricular dysfunction [145]. Further exploration of this drug, along with other complement inhibitors, could prove useful in future treatments.

The current treatment options for PH vary for each patient and subtype and can improve patient prognosis and/or symptoms. In the case of PH due to left heart disease, treatments primarily target the underlying cardiac condition. Left heart failure commonly involves excessive stimulation of the renin–angiotensin–aldosterone system (RAAS), as well as adrenergic activation. Renin can induce complement activation in the alternative pathway, and drugs like ACE inhibitors and ARBs, which inhibit the RAAS, were recently associated with significantly increased C4 levels in patients with IgA nephropathy [146]. Management therapies include medications such as beta-blockers, angiotensin enzyme converting (ACE) inhibitors, angiotensin receptor blockers (ARBs), angiotensin receptor/neprilysin inhibitors (ARNIs), and aldosterone blockers [147]. PH due to lung disease is most often treated with supplemental oxygen, especially in cases with underlying COPD [70]. In manifestations of the condition precipitated by ILDs, such as IPF, the antifibrotic agents pirfenidone and nintedanib may augment care [148]. An existing work suggests that crosstalk between TGF-β1 and complement contributes to and augments epithelial injury in IPF [149]. Given that pirfenidone can decrease the expression of TGF- β, it may have an indirect role in modifying complement activity. Nintedanib also likely indirectly affects complements via its inhibition of platelet-derived growth factor (PDGF), which can activate complements via both classic and alternative pathways [150]. Chronic thromboembolic pulmonary hypertension (CTEPH) is treated both surgically and medically, with anticoagulants followed by curative pulmonary endarterectomy or pulmonary balloon angioplasty in eligible patients. Patients who are ineligible for surgical intervention may receive riociguat, a stimulator of soluble guanylate cyclase (sGCS), or any of many other PAH-targeted drugs that have yet to receive Food and Drug Administration (FDA) approval for CTEPH but may show promising effects [151,152,153,154]. Combination therapy with the five accepted classes of PAH medications is the standard of care for PAH treatment, and individual therapies depend on the patient’s 1-year mortality risk [155,156]. Notably, the pathophysiological mechanisms include complement and coagulation pathways in the CTEPH population, as measured using serum proteomics analysis [157]. The most commonly prescribed combination involves concurrent phosphodiesterase type 5 (PDE-5) inhibitor and endothelin receptor antagonist (ERA) use. These drugs do not directly interact with the complement, but PDE-5 inhibitors act via the aforementioned NO/sGC pathway to downregulate inflammation. They are also currently being investigated for possible immunomodulatory effects in autoimmune conditions such as rheumatoid arthritis [158,159]. Endothelin receptor antagonists are a commonly prescribed treatment for PAH patients [160]. Endothelin-1 is a peptide vasoconstrictor produced from endothelial cells, vascular smooth muscle cells, the renal medulla, and macrophages. It binds with G-protein coupled endothelin-1 ETA and ETB receptors on effector cells and stimulates contraction of the tunica media smooth muscle layer in vessel walls. Endothelin-1 also has pro-fibrotic and pro-inflammatory effects [161,162,163,164]. Phosphodiesterase type 5 inhibitors such as sildenafil are another commonly prescribed treatment for PAH. They stimulate vasodilation by inhibiting the breakdown of cyclic guanosine monophosphate (cGMP). This intracellular secondary messenger molecule regulates smooth muscle cell relaxation and prolongs the effects of vasodilators such as nitric oxide (NO) [165,166]. Soluble guanylate cyclase is an enzyme in the nitric oxide (NO) signaling pathway that facilitates vasorelaxation and subsequent dilation by binding NO and catalyzing the synthesis of cGMP. Soluble guanylate cyclase stimulators are useful in treating PAH, because they enhance NO signaling in the pulmonary arteries [167,168]. Prostacyclin analogs such as epoprostenol and treprostinil and prostacyclin receptor analogs such as selexipag comprise the final category of drugs currently used for the treatment of PAH. Prostacyclin is a hormone released from vascular endothelial cells that mediates localized inflammation and stimulates vasodilation by relaxing smooth muscle [169,170,171,172,173]. Prostacyclin and pentraxin 3 modulate inflammation with contrasting effects in chronic inflammation and in the development of PAH. Pentraxin 3 induces endothelial inflammation and enhances blood clotting, which could be modulated by its direct interactions with the complement factors C1q and C3b and factor H [174,175,176,177,178]. Prostacyclin acts as a vasodilator by promoting vascular health and blocks platelet aggregation, which are often impaired in PAH patients by potentially impacting on infiltrating immune cells and complement components. A 2023 study also investigated the value of nonpharmacological therapy by examining the effect of pulmonary rehabilitation on vascular repair mechanisms in patients with stable PAH; endurance training was associated with positive effects and repair promotion [179]. Calcium channel blockers (CCBs) are another treatment option used primarily for a small subset of patients with idiopathic PAH. They lower blood pressure by reducing calcium ion influx to smooth muscle cells to initiate vasodilation. Only about 5% of the PAH population responds well to long-term treatment with calcium channel blockers, and the likelihood of a positive response is determined by pulmonary vasoreactivity testing [180,181,182,183]. Although new work on the relationship between CCBs and complement remains sparse, a 1988 study indicated that, while verapamil does not directly affect C3a levels in the hemodialysis population studied, nifedipine and verapamil inhibited granulocyte activation in the context of reduced levels of complement signaling [184]. Additionally, many pro-inflammatory activities of polymorphonuclear leukocytes (PMNLs) such as chemotaxis and other functional responses depend on calcium ion mobilization [185]. Sotatercept is another PAH treatment that recently received FDA approval in March of 2024, as discussed in a later section. The current data show the effectiveness of this treatment in experimental sugen–hypoxia rat models [186]. Trials in humans have also demonstrated positive results, including decreased pulmonary vascular resistance and improved exercise capacity when used in combination therapy for PAH [187,188]. Therefore, approved treatment options for PH extend patients’ life expectancy and treat disease symptoms. However, there are no available pharmacologic treatment options for PH that directly prevent or reverse disease development. The current treatments for PH primarily act on biochemical pathways that lead to arterial vasodilation, and treatments targeted at pathophysiological mechanisms that lead to the development of vascular remodeling should be the focus of future research.

## 9. Completed and Ongoing Clinical Trials in PAH

An account of the completed and ongoing human clinical trials in PAH are detailed in Table 1. Research into effective therapeutics for PAH is continually evolving. Recent clinical trials fall primarily into two categories: those assessing the safety and ideal dosing of new drugs and those investigating the utility of accepted or promising medications in combination therapy.

One ongoing study proposes that empagliflozin, a sodium-glucose cotransporter 2 (SGLT2) inhibitor commonly used to treat diabetes, may prove effective in treating PAH in humans. This proposition is undergirded by evidence that, in rats with (MCT)-induced PAH, empagliflozin reduces mortality, decreases right ventricle systolic pressure, and attenuates pulmonary remodeling (Study #1) [189]. Additionally, multiple recent studies investigating the relationship between SGLT2 inhibitors and complement suggest that empagliflozin and dapagliflozin attenuate and mediate complement-influenced disease processes in murine models of kidney disease, which holds intriguing implications for empagliflozin as a possible treatment agent in PAH [190,191]. The 2018 TRANSFORM-UK therapeutic open-label study assessed the benefit of tocilizumab, a monoclonal antibody that binds IL-6 and has disease-modifying properties in rheumatoid arthritis. Tocilizumab decreases the serum C3 and C4 complement protein levels in rheumatoid arthritis [192], which may benefit to block the progression of PAH. While the results indicated an overall decrease in c-reactive protein (CRP) in treated individuals with PAH, pulmonary vascular resistance remained unaffected, and right ventricle function was not measured (Study #5) [193]. Although researchers observed no effect on pulmonary vascular resistance over six months of treatment in this trial, the sample size was small, and the findings should not preclude further investigation of tocilizumab. A recently completed trial of the experimental drug CS1 examined its safety and efficacy at different doses in PAH patients (Study #6). CS1 is a controlled-release form of valproic acid that acts as a histone deacetylase (HDAC) inhibitor, and the study will assess the change from baseline in patients who received a 12-week treatment course. Valproic acid has a previously demonstrated effect in the downregulation of hyperglycemia-induced complement pathways and attenuates cellular senescence in murine models of diabetes via C5a receptor inhibition [194,195]. The APPROACH-2 study intends to examine the utility of the bromodomain and extraterminal (BET) protein inhibitor apabetalone (Study #4). Existing research suggests that apabetalone attenuates vascular inflammation and reduces the risk of adverse cardiac events in patients with cardiovascular disease, and researchers posit that this mechanism may benefit individuals with PAH [196]. Additionally, apabetalone has been demonstrated to downregulate the complement cascade widely and block the induction of complement in both preclinical and human studies [196,197,198]. The tyrosine kinase inhibitor imatinib is also an intriguing option for PAH and can occasionally be offered on a compassionate basis to patients for whom other treatment options have been exhausted. While no current work specifically examines the effect of imatinib on complement in humans, some known tyrosine kinases contribute to complement-mediated phagocytosis [199]. Complement has also been implicated in shaping the tumor microenvironment of certain cancers and influencing sensitivity to tyrosine kinase inhibitors [200]. The phase 2 PIPAH study plans to assess the validity of concerns regarding the safety and tolerability of imatinib in PAH patients (Study #7).

The recently completed INSPECTIO observational study enrolled individuals with diagnosed PAH on either monotherapy or combination therapy regimens, which included FDA-approved PAH treatments macitentan or selexipag (Study #8). Macitentan antagonizes the endothelin receptor subtypes ETA and ETB, while selexipag is a prostacyclin receptor agonist. Researchers measured changes from baseline over 12 months in study participants via the following risk criteria: World Health Organization Functional Class (WHO/FC), 6-min walk distance (6MWD), brain natriuretic peptide (BNP), and NT-proBNP. The upcoming publication will compare changes between individuals receiving macitentan versus selexipag-containing regimens. Endothelin and complement receptors are commonly upregulated during times of local or systemic inflammation, with one study of placental ischemia suggesting a complement-modulating role for endothelin [201]. Quantities of serum complement in the population differed from those expressed locally, implying that this modulatory effect might be more pronounced with regional endothelin signaling. With respect to the mechanism of selexipag, data on the relationship between complement and prostacyclin remain sparse. A 2015 Phase I trial examined the safety and hemodynamic effects of the antianginal drug ranolazine in PAH patients on monotherapy or dual therapy treatment courses (Study #9). While ranolazine appeared safe for use at the tested dosage, only a small group of participants achieved therapeutic levels [202]. The drug sotatercept, which binds to and inhibits activin class proteins within the transforming growth factor beta (TGF-β) superfamily, received FDA approval in March 2024 for treating PAH. Recent trials have focused on its pharmacokinetics, as well as safety and efficacy in patients on background PAH therapy (Study #2, Study #3, and Study #10). No current work examines the relationship between sotatercept and complement, but the drug received expedited approval and has yet to be studied extensively in concert with inflammatory pathways other than those directly implicated in its mechanism.

## 10. Preclinical Studies on Therapeutics Targeting Complement in PAH

The development of therapies targeting the complement system’s role in PAH represents an emerging frontier in treatment. Recent studies have focused on therapies designed to inhibit the initial components of the complement cascade, with promising results demonstrated in preclinical models. In one such study, a novel compound called CP40-KK, an analog of the C3 inhibitor CP40, was tested in a rat model of PAH. Both experimental groups received MCT to induce PAH, with one group treated with CP40-KK while the other served as a control. CP40-KK, a fourth-generation compstatin (Amyndas Pharmaceuticals) known to inhibit C3, reduces downstream complement activation. Rats treated with CP40-KK exhibited reduced NLR family pyrin domain-containing protein 3 (NLPR3) inflammasome activation, less pulmonary vascular remodeling, and decreased right ventricular hypertrophy compared to the controls. These protective effects were attributed to a decrease in C3a levels, which, in turn, reduced the activity of pro-inflammatory cytokines IL-1β and interleukin-18 (IL-18) [203]. This study underscores the potential of complement-targeted therapies in mitigating complement-mediated vascular remodeling, highlighting a promising direction for future research in PAH treatment. Indeed, the intraocular delivery of compstatin CP40-KK and another other analog, CP40-KKK, have significantly protected against age-related macular degeneration in non-human primate models [204].

## 11. Future Insights for Complement in Pulmonary Hypertension and Other Diseases

Dysregulation of a complement is defined as either insufficient activation or hyperactivation of the different complement pathways and is associated with a diverse spectrum of diseases [103,205]. The complement system directly affects the inflammatory mechanisms in lung tissue, including but not limited to macrophage recruitment, pro-inflammatory cytokine production, fibroblast stimulation, and mast cell stimulation [50]. Even more emerging data show that anaphylatoxins regulate gene transcription for pro-inflammatory cytokines in endothelial cells. Further research on this mechanism in lung tissue could improve our understanding of vascular changes in the pulmonary arteries in PH.

Within the past several years, a few studies examined PAH phenotypes. The observational cohort study done at Stanford University was able to distinguish PAH immune phenotypes by using machine learning to examine blood cytokine levels [206]. Another observational cohort study done in London examined patients with PAH and found a link between plasma protein markers and mortality risk for those with PAH [207]. This technology could prove useful in earlier disease identification and more accurate prognosis determination in PAH patients.

PH is still considered a rare disease, but there is evidence to suggest that the prevalence of this disease could increase in future generations [208]. Currently, around 50% of Americans suffer from some form of hypertension [209]. Proper functioning of the pulmonary artery and pulmonary vascular bed is critical to maintain cardiovascular homeostasis. Emerging research on murine models and human lung tissue samples shows direct reliance on complement mechanisms in localized inflammation and vascular remodeling processes [65]. Due to insufficient data and research, a complement’s involvement in PH was considered speculative until recently. Traditionally viewed as a mere “complement” to the innate and adaptive immune responses, the complement system is much more complex than originally believed. Future research could provide useful data on the effectiveness of complement inhibitors or antagonists of related pathways in preventing deleterious vascular changes associated with PH development and progression.

## 12. Conclusions and Future Directions

Pulmonary hypertension is a deadly and widespread disease that causes the often fatal thickening of arterial vessels and increased blood pressure. Despite the presence of numerous drugs for treating a range of PH conditions, there is no cure, and most who have it die within 2–3 years. However, recent findings have shown a direct association between the complement system and the development of pulmonary hypertension, which could offer new avenues for treatment.

Chronic inflammation is among the most prominent mechanisms associated with vascular remodeling. The complement system, activated by any of its three pathways, is responsible for producing anaphylatoxins like C3a and C5a, which bind to endothelial cells, mast cells, macrophages, and other leukocytes to generate a multifaceted pro-inflammatory response, including the release of cytokines and chemokines like IL-1α, IL-6, IL-8, TNF, and oncostatin M, as well as histamine. In turn, each of these cytokines possesses their own pro-inflammatory pathways, whether through gene transcription effects, regulation of acute phase pro-inflammatory proteins, activation and attraction of neutrophils and macrophages, remodeling of the ECM, or differentiation of fibroblasts into myofibroblasts. All of these processes ultimately contribute to a broad, cascading inflammatory response [33,49,50,51,52]. Anaphylatoxins have also been shown to mediate smooth muscle cell contraction, a form of biomechanical stress resulting in vascular smooth muscle cell hypertrophy [45,46,47,48]. Overall, these findings point to complement protein anaphylatoxins as effectors of vascular remodeling in the pulmonary arteries.

Within PAH specifically, a recent study found a link between the complement system cascade and hypoxia-induced PH, reporting C3 deposition within minutes of mouse exposure to hypoxic environments. Notably, hypoxic environments resulted in the deposition of IgM and IgG antibodies, resulting in the antibody-dependent activation of the complement system and subsequent pro-inflammatory response associated with vascular remodeling [65]. The complement system may also play a role in the subtypes of PH associated with COPD, IPF, and ILDs, such as Group 3 PH. This is indicated by the activity of TNF, which is released when anaphylatoxins bind macrophages in various lung-related diseases like COPD and lung cancer [50,75]. It is also indicated by high levels of complement protein C1q and myofibroblasts, which are differentiated from fibroblasts by IL-6, in patients with pulmonary fibrosis [77,78].

Evidence has also pointed to circadian rhythm effects on localized vascular remodeling. Interestingly, the reported mechanisms possess overlapping components with complement system pathways: BMAL1-deficient lung fibroblasts exhibit higher CXCL5 expression when stimulated with IL-1β [121], mice deficient in circadian rhythm genes *CRY1* and *CRY2* have elevated levels of IgG and IgM that activate the complement system classical pathway [126], and the platelet function becomes dysregulated [134]. As such, the circadian rhythm also warrants further research to elucidate possible associations with the complement system and their broader role in the onset and progression of PH.

Taken altogether, the aforementioned research has offered promising evidence to support the influence of complement and circadian rhythm mechanisms on localized inflammation and vascular remodeling. Further studies should be conducted to explore the efficacy of therapies targeting specific components of complement system pathways in mitigating adverse vascular transformations as they relate to PH. Moreover, the use of improved diagnostic methods, such as machine learning and plasma protein markers, could enhance the accuracy of prognoses and potentially offer more favorable outcomes associated with this deadly disease.

## Figures and Tables

**Table 1:** Ongoing and Completed Clinical Trials on Pulmonary Arterial Hypertension.

No.	Study Title	Conditions	Interventions	Clinical Trial Number	Status
1	A Study on the Efficacy and Safety of Empagliflozin in the Treatment of Pulmonary Arterial Hypertension	Pulmonary arterial hypertension	Drug: Empagliflozin Other: Placebo	NCT06554301	Ongoing
2	A Clinical Study of Sotatercept (MK-7962) in People with Pulmonary Arterial Hypertension (LIGHTRAY)	Pulmonary arterial hypertension	Drug: Sotatercept	NCT06664801	Ongoing
3	Right Ventricular Compensation with Sotatercept: a Prospective Single Arm Open Label Phase 4 Study to Evaluate the Effects of Sotatercept on Right Ventricular Function in Pulmonary Arterial Hypertension (RECOMPANSE)	Pulmonary arterial hypertension	Drug: Sotatercept	NCT06658522	Ongoing
4	Apabetalone for Pulmonary Arterial Hypertension (APPROACH-2)	Pulmonary arterial hypertension	Drug: Apabetalone Other: Placebo	NCT04915300	Ongoing
5	A Therapeutic Open Label Study of Tocilizumab in the Treatment of Pulmonary Arterial Hypertension (TRANSFORM-UK)	Pulmonary arterial hypertension	Drug: Tocilizumab	NCT02676947	Completed
6	Effect of CS1 in Patients With Pulmonary Arterial Hypertension	Pulmonary arterial hypertension	Drug: CS1	NCT05224531	Completed
7	Positioning Imatinib for Pulmonary Arterial Hypertension (PIPAH)	Pulmonary arterial hypertension	Drug: Imatinib Mesylate	NCT04416750	Completed
8	A Study of Pulmonary Arterial Hypertension Participants Treated With Macitentan or Selexipag	Pulmonary arterial hypertension	Drug: Macitentan, Selexipag	NCT04567602	Completed
9	A Study of Ranolazine Acute Administration and Short Term Administration in Pulmonary Arterial Hypertension	Pulmonary arterial hypertension	Drug: Ranolazine Other: Placebo	NCT01757808	Completed
10	A Study of Sotatercept for the Treatment of Pulmonary Arterial Hypertension (STELLAR)	Pulmonary arterial hypertension	Drug: Sotatercept Drug: Background PAH therapy Other: Placebo	NCT04576988	Completed
